# Dementia in Latin America: Epidemiological Evidence and Implications for Public Policy

**DOI:** 10.3389/fnagi.2017.00221

**Published:** 2017-07-13

**Authors:** Nilton Custodio, Ana Wheelock, Daniela Thumala, Andrea Slachevsky

**Affiliations:** ^1^Unidad de diagnóstico de deterioro cognitivo y prevención de demencia, Departamento de Neurología, Instituto Peruano de Neurociencias Lima, Peru; ^2^Gerosciences Center for Brain Health and Metabolism (GERO) Santiago, Chile; ^3^Department of Surgery and Cancer, National Institute for Health Research Imperial Patient Safety Translational Research Centre, Imperial College London London, United Kingdom; ^4^Psychology Department, Faculty of Social Sciences, Universidad de Chile Santiago, Chile; ^5^Physiopathology Department, ICBM, and East Neuroscience Department, Faculty of Medicine, Universidad de Chile Santiago, Chile; ^6^Cognitive Neurology and Dementia Unit, Neurology Department, Hospital del Salvador Santiago, Chile; ^7^Center for Advanced Research in Education (CIAE), Universidad de Chile Santiago, Chile; ^8^Servicio de Neurología, Departamento de Medicina, Clínica Alemana-Universidad del Desarrollo Santiago, Chile

**Keywords:** Latin America, dementia, epidemiology, public policy, Alzheimer, dementia plan, caregiver burden, cost of dementia

## Abstract

Population aging is among the most important global transformations. Today, 12% of the world population is of age 60 and over and by the middle of this century this segment will represent 21.5%. The increase in population of those aged 80 and over, also referred to as the “oldest old” or the “very elderly”, will be even more pronounced, going from 1.7% of the population to 4.5% within the same period. Compared to European and North American countries, Latin America (LA) is experiencing this unprecedented demographic change at a significantly faster rate. Due to demographic and health transitions, the number of people with dementia will rise from 7.8 million in 2013 to over 27 million by 2050. Nowadays, the global prevalence of dementia in LA has reached 7.1%, with Alzheimer’s Disease (AD) being the most frequent type. This level is similar to those found in developed countries; however, the dementia rate is twice as high as that of the 65–69 years age group in developed countries. In addition, the prevalence and incidence of dementia is higher among illiterate people. Mortality rates due to dementia have risen considerably. The burden and costs of the disease are high and must be covered by patients’ families. The prevention of dementia and the development of long-term care policies and plans for people with dementia in LA, which take into account regional differences and similarities, should be urgent priorities.

## Introduction

Population aging is among the most important global transformations. Today, 12% of the world population is of age 60 and over and by the middle of this century this segment will represent 21.5%. The increase in population of aged 80 and over, also referred as the “oldest old” or the “very elderly”, will be even more pronounced, rising from 1.7% of the population to 4.5% within the same period (United Nations, 2015). Compared to European and North American countries, Latin America (LA) is experiencing this unprecedented demographic change at a significantly faster rate (Bongaarts, 2009). Due to demographic and health transitions, the number of people with dementia in LA will rise from 7.8 million in 2013 to over 27 million by 2050 (Bupa and Alzheimer’s Disease International, 2013; Baez and Ibáñez, 2016). The aim of this review is to provide an overview of the epidemiological state of affairs and a critical appraisal of the public policies that are being designed/implemented vs. those that are needed to address the current dementia-related challenges.

## The Prevalence of Dementia in Latin America

As a result of the demographic transition in LA, the total number of individuals over 60 years of age will increase, reaching approximately 57 million by 2025 (CEPAL, 2014). On the other hand, low socioeconomic and educational levels in the region are additional elements that account for the major increase in the prevalence of dementia. For these reasons, dementias are beginning to be regarded as a public health priority. Systematic reviews of studies on the prevalence of dementia conducted in developed countries have revealed a slightly downward trend for the USA and Europe, but an upwards trend for Asia (Winblad et al., 2016), The analysis of eight population studies conducted in Brazil, Cuba, Chile, Peru and Venezuela showed that the global prevalence is 7.1% (confidence interval, CI 95%: 6.8–7.4; Nitrini et al., 2009). The studies reveal that the prevalence of dementia increases with age, doubling every 5 years from 65 years of age onwards. It rises from 2.40% (CI 95%: 2.11–2.72) in the 60–64 group to 33.07% (CI 95%: 29.98–36.20) in the 90–94 group (Nitrini et al., 2009). However, there is a significant variation in the estimate of prevalence of dementia, ranging from 2% in a Brazilian study (Ramos-Cerqueira et al., 2005) to 13% in a Venezuelan study (Maestre et al., 2002). These studies suggest prevalence similar to the ones reported for developed regions, whose rates range from 4.2% in Canada to 14.5% in Spain (Winblad et al., 2016). We must stress the fact that at least three studies, those conducted in Catanduva-Sao Paulo (Herrera et al., 2002; Nitrini et al., 2004) and Lima (Custodio et al., 2008), used the same screening tests for diagnosing dementia, while Llibre’s study in Cuba (Llibre et al., 1999) used a uniform structured interview (Table 1).

**Table 1:** Prevalence of dementia: community-based studies 1994–2000 (Lopes et al., 2007).

Age	Number of studies	Prevalence of dementia (%) (CI** 95%)	Average increase in prevalence
65–69	17	1.2 (0.8–1.5)	—
70–74	19	3.7 (2.6–4.7)	3.0
75–79	21	7.9 (6.2–9.5)	2.1
80–84	20	16.4 (13.8–18.9)	2.0
85–89	16	24.6 (20.5–28.6)	1.5
90–94	6	39.9 (34.4–45.3)	1.6
>95	6	54.8 (45.6–63.9)	1.3

**CI, confidence interval.

With respect to the types of dementia found in the Brazilian city of Catanduva, 25% of the participants were assessed in their urban homes at ≥65 years of age. Specialists examined those with suspected dementia. A 7.1% rate of dementia was found, with 55.1% of Alzheimer’s Disease (AD) cases (Herrera et al., 2002). A similar protocol was used in Lima with a sample of 1532 individuals. 105 cases of dementia were found, with AD being the most frequent diagnosis (56.2%). This prevalence increased with age and was mostly observed in female participants (Custodio et al., 2008). AD is the most frequent cause of dementia in LA, representing 56.3% of cases, followed by AD with cerebrovascular disease (CVD), which reaches 15.5%, and vascular dementia (VD), which accounts for 8.7%. These results are consistent with those reported in previous research (Herrera et al., 2002; Nitrini et al., 2004; Ramos-Cerqueira et al., 2005). However, the fact that magnetic resonance imaging was not used may have distorted the true proportion of individuals with AD+CVD and VD (Grinberg et al., 2007; França-Resende et al., 2016).

Few studies conducted in our region have focused on other degenerative causes of dementia, such as frontotemporal dementia (FTD). Community-based studies conducted in LA with individuals aged ≥55 years have found that the prevalence of FTD ranges from 12 to 18 cases per 1000 people, and that it is higher among Brazilians (2.6%–2.8%; Herrera et al., 2002; Lopes, 2006) than among Peruvians (1.90%; Custodio et al., 2008) and Venezuelans (1.53%; Maestre et al., 2002; Table 2). The values reported are intermediate with respect to global studies (Custodio et al., 2013). In general, the exact prevalence of FTD is unknown. The prevalence estimated in studies with outpatients and in European memory centers ranges from 0.002% to 0.031%; from 0.078% to 1.56%, and from 0.054% to 0.135% between 45 years and 64 years, 65 years and 74 years, and at ≥75 years respectively (Rosso et al., 2003; Gilberti et al., 2012). However, recent community-based studies suggest that FTD may be more common than previously estimated (Bernardi et al., 2012). Borroni’s (Borroni et al., 2010) in Brescia, Italy, reported a higher prevalence than the literature, reaching 17.6 cases per 100,000 people. This study encourages us to pay attention to FTD in people over 75 years old, in whom the prevalence reached 54/100,000 inhabitants. The research examined suggests that the prevalence of FTD in LA may be lower than that reported for developed countries.

**Table 2:** Prevalence of frontotemporal dementia in four studies conducted in Latin America (LA; adapted from Custodio et al., 2013).

Country	Location	Age group	Prevalence			Causes of dementia	
			Global dementia (95% CI^a^)	FTD^b^	DUE^c^	FTD (%)	DUE (%)
Brazil (Herrera et al., 2002; Nitrini et al., 2004)	Catanduva, Sao Paulo	≥65	7.1% (6.0–8.5)	0.18%	0.90%	2.60	12.70
	Ribeirao Preto, Sao Paulo	≥60	6.0% (4.6–7.3)	0.7%	0.43%	2.80	7.20
		≥65	7.2% (5.7–8.6)	–	–	–	–
Venezuela (Maestre et al., 2002)	Santa Lucia, Maracaibo	≥55	8.04% (7.01–9.19)	0.12%	0.45%	1.53	5.61
		≥65	13.27%^†^	–	–	–	–
Peru (Custodio et al., 2008)	Cercado de Lima, Lima	≥65	6.85% (5.53–8.08)^‡^	0.13%	0.87%	1.90	12.70

*^a^CI, confidence interval; ^b^FTD, fronto-temporal dementia; ^c^DUE, dementia of unknow etiology; ^†^data calculated by author; ^‡^confidence interval data provided by author. Only raw, non-adjusted data are presented*.

## The Incidence of Dementia in LA

In the study conducted in Catanduva, Sao Paulo (Nitrini et al., 2004), 1119 individuals aged ≥65 years were reassessed on average 3.25 years after their first assessment, a process that revealed 50 cases of dementia (28 AD cases). The incidence rate of dementia was 13.8 per 1000 people/year for individuals ≥65 years, while that of AD was 7.7. This is comparable to the levels reported by researchers working in Europe, North America and Asia (Satizabal et al., 2016). The incidence rate of dementia doubled every 5 years; however, it was found to be lower in the last 5-year period studied (Table 3). This may be due to the number of individuals in the age group over 90 years (*n* = 16), where only two new cases of dementia were found. This phenomenon has also been observed in other studies (Satizabal et al., 2016). In contrast with prior research, the study conducted in Catanduva (Nitrini et al., 2004) did not demonstrate any significant differences linked to gender; however, female participants displayed a high incidence of dementia, especially AD, in very elderly groups (over 85 years of age). The incidence of dementia was higher in males than in females in the group ≤85 years, although this difference was not statistically significant. The study carried out by the 10/66 Dementia Research Group with individuals ≥60 years old living in urban areas of Cuba, the Dominican Republic and Venezuela and in urban and rural areas of Peru, Mexico and China (Prince et al., 2013) reported a higher incidence rate of dementia in women than in men and revealed that it increased exponentially with age. After standardizing the EURODEM incident cohort for age (analysis of four prospective studies conducted in Denmark, France, Netherlands and United Kingdom), the incidence rate of dementia according to 10/66 criteria ranged from 20 to 30 per 1000 people/year, which is slightly higher than the 18.4 per 1000 people/year calculated according to the DSM-III-R criteria reported by the EURODEM. On the other hand, the incidence rates of dementia according to 10/66 criteria were approximately 1.5–2.5 times higher than those obtained with the DSM-IV dementia criteria.

**Table 3:** Rate of incidence of dementia by age group and sex per 1000 inhabitants/year (adapted from Nitrini et al., 2004).

Age	*N*	Women (CI** 95%)	Men (CI 95%)	Total (CI 95%)
65–69	2	2.4 (0.52–0.725)	3.9 (0.845–11.60)	3.0 (0.4–10.7)
70–74	9	4.0 (1.74–7.77)	9.3 (5.72–15.05)	6.4 (3.0–12.1)
75–79	14	13.7 (9.36–23.87)	20.5 (14.04–32.92)	16.4 (9.1–27.1)
80–84	10	19.2 (3.87–32.05)	35.8 (21.12–58.66)	25.0 (12.2–44.4)
85–89	13	68.4 (5.13–98.60)	24.3 (11.64–45.83)	48.2 (26.5–77.8)
≥90***	2	44.0 (9.46–113.46)	34.2 (7.38–91.74)	38.5 (4.8–118.0)

**CI, confidence interval, ***Only one patient (97) was over 94 years old.

## Risk Factors Associated with Dementia

### The Influence of Age and Gender on the Prevalence of Dementia in LA

According to studies conducted in LA, the prevalence of dementia increases with age: from 2.40% (95% CI: 2.11–2.72) in the 65–69 group to 20.20% (95% CI: 18.62–21.78) in the 85–89 group and 33.07% (95% CI: 29.98–36.20) among participants aged 90–94 years (Nitrini et al., 2004). These results confirm that the prevalence rates of dementia increase exponentially with age (Prince et al., 2016; Winblad et al., 2016).

In terms of gender, studies conducted in LA show higher rates for both men and women in the 65–69 years group, and for women in the 70–74 years group, compared with the results reported by European studies (Lobo et al., 2000; Nitrini et al., 2004; Winblad et al., 2016; Table 4). The studies carried out in LA reported slightly higher rates for female participants in all age groups (Nitrini et al., 2004). Similar rates were reported in studies conducted in Europe (Lobo et al., 2000; Winblad et al., 2016), LA, India and China (Prince et al., 2013). It is interesting to note that in LA, the prevalence in the 65–69 group is higher than in developed countries (Table 4). A number of reasons may be contributing to the greater prevalence observed in relatively young individuals in developing countries, especially their limited access to primary care and their low educational level. The lack of primary health care can predispose these individuals to suffering from dementia caused by controllable or curable diseases, such as hypertension or syphilis. Low educational levels have been consistently linked to high dementia rates; in this regard, it can be argued that low educational levels are linked to early manifestations of cognitive decline, whereas individuals with higher educational levels tend to have a cognitive reserve that delays the emergence of clinical signs of dementia (Fratiglioni and Wang, 2007; Manly et al., 2007).

**Table 4:** Comparison of dementia prevalence associated with gender considering data provided by seven LA studies and European studies (adapted from Nitrini et al., 2009).

	Latin American studies						European studies	
	Women			Men			Women	Men
Age	Dementia *N*	Participants *N*	Mean (%) (95% CI**)	Dementia *N*	Participants *N*	Mean (%) (95% CI)	Mean (%)	Mean (%)
65–69	149	5620	2.65	79	3479	2.27	1.0	1.6
			(2.25–3.10)			(1.80–2.81)		
70–74	196	4781	4.10	65	2317	2.81	3.1	2.9
			(3.55–4.69)			(2.17–3.57)		
75–79	293	3802	7.71	112	1888	5.93	6.0	5.6
			(6.89–8.59)			(4.90–7.09)		
80–84*	291	2326	12.51	162	1489	10.88	12.6	11.0
			(11.17–13.94)			(9.34–2.55)		
85–89	281	1244	22.59	182	960	18.96	20.2	12.8
			(20.30–24.97)			(16.49–21.55)		
90+	189	500	37.80	105	390	26.92	30.8	22.1
			(33.56–42.28)			(22.54–31.67)		

**CI, confidence interval.

### The Influence of Education on Dementia

An inverse relationship has been demonstrated to exist between educational level and dementia. The prevalence of dementia in Lima reached 3.7% in individuals with more than 8 years of education, but it was much higher (15.2%) in illiterate participants (Custodio et al., 2008). Through a univariate analysis, the study conducted in Catanduva also revealed a greater incidence of dementia among illiterate participants; however, a multivariate analysis showed that the relationship was more significant when controlling for age and female sex (Nitrini et al., 2004). In Chile, two studies reported higher prevalence of cognitive impairment and dementia in rural contexts and in people with low educational levels: cognitive impairment was 5.6 times higher among adults with low educational levels (17.2%) compared to those with high educational levels (3%; González et al., 2009; ENS, 2010; Fuentes and Albala, 2014).

The systematic review published by Sharp and Gatz (2011), which involved 71 studies conducted in the last 25 years, suggests that the education-dementia link may be more complex. This may be due to the fact that differences in terms of the methodologies and samples used make it difficult to compare studies; however, the analysis of the educational factor could have a differential impact in different cultures and cohorts. The mechanisms that have been advanced to explain the education-dementia link include cerebral reserve and cognitive reserve (Stern, 2009), which appear to be robust. Likewise, there is consensus among researchers that education is a protective factor against dementia (Caamaño-Isorna et al., 2006; Baumgart et al., 2015). Within the context of the cognitive reserve hypothesis, if two individuals with the same brain volume suffer from dementia, the one with the largest cognitive reserve is believed to be better equipped to tolerate the cerebral pathological burden for longer, thus delaying the first clinical manifestations of the disease (Qiu et al., 2001). In addition, the survival rates of patients with dementia and high education levels are low compared with those of patients with low education levels (Qiu et al., 2001). Stern’s theoretical explanation (Stern, 2012) is that, when individuals with high cognitive reserves reach the point where cognitive function is affected, they have a cerebral pathological burden nearing their total capacity, and at that point clinical manifestations are florid and evolve quickly, which resembles rapidly progressive dementia. On the other hand, it is interesting to trace the influence of non-modifiable risk factors such as allele ε4 of apolipoprotein E (APOE) and its effect considering education level. In this regard, there are no doubts of the role of APOE ε4 as a risk factor for AD; however, education may be relevant for balancing the effect of APOE ε on the clinical manifestations of dementia. Only a few studies have been conducted on the latter issue. An analysis of the data yielded by three studies conducted in Northern Europe (Sweden and Finland) with 3436 participants over 65 years old suggests that genetic factors (APOE ε4) and environmental factors (education) may act independently as risk factors for dementia; in addition, they may interact: high education levels could balance the negative effects of APOE ε4 on the occurrence of dementia (Wang et al., 2012). There is no information about the influence of education and Apo-E on AD.

## Dementia-Related Mortality

People living with dementia have a risk of death 2–4 times higher than that of similarly aged people without dementia (Ientile et al., 2013). In middle-income countries, the mortality risk is 1.56–5.69 times higher than in individuals without dementia (Nitrini et al., 2005). Survival after a dementia diagnosis ranges from 3 years to 12 years, depending on diagnostic criteria, age, severity at the time of diagnosis, and place of diagnosis (Brodaty et al., 2012; Kua et al., 2014). Fitzpatrick et al. (2005) reported an average survival rate of 7.1 years (95% CI: 6.7–7.5 years) for AD and 3.9 years (3.5–4.2 years) for VD. Helzner et al. (2008), using a multi-ethnic cohort of 323 individuals in the USA, described that AD reduced life expectancy by 3 years in subjects diagnosed between 70 years and 75 years of age, and by 1–2 years when diagnosis was made at a later age. In advanced dementia cases, average survival reached 1.3 years, with a mortality rate of 25% at 6 months. Similarly, Mitchell et al. (2009), with a cohort of 323 nursing home patients with advanced dementia, report an average survival time of 1 year and 3 months and a mortality risk of 24.7% at 6 months. At 18 months of evolution, more than 54.8% of patients had died. Severe dementia often causes complications such as immobility, swallowing disorders and malnutrition, thus increasing the risk of developing intercurrent diseases that may cause death. It has been reported that in the last 3 months of survival, 37.3% of patients suffered from pneumonia, 32.2% had a fever, and 90.4% displayed swallowing disorders. Pneumonia has been identified as the complication most frequently leading to death (Mitchell et al., 2009; for a review, see Slachevsky et al., 2016).

A more than threefold increase in dementia-related mortality was observed between 1990 and 2010; thus, the number of deaths went up from 141,200 (CI 95% 110,800–208,500) to 485,700 (CI 95% 307,800–590,500), which represents a 244% increase. The age-adjusted mortality rate rose from 3.6 (CI 95% 2.8–5.4) to 7.1 (CI 95% 4.5–8.6) per 100,000 inhabitants, which constitutes a 95.4% increase. Globally, AD and other dementias are the 50th cause of death (Prince et al., 2016). In the Andean part of LA, they are the 68th cause and the 53th in South America, with major intra-regional disparities (Lozano et al., 2012; Prince et al., 2016). In Chile, in 2012, with 3852 deaths (3.89% of the total number of deaths), dementias constituted the sixth specific cause of death (Lozano et al., 2012). A global study on disease burden conducted in 2010 showed that the number of deaths attributed to dementias rose by 526%, which means that they are the cause of death with the largest increase in percentage (Lozano et al., 2012). In Chile, regional variability has been reported with respect to dementia-related mortality. Two regions display higher rates: the Metropolitan Region, in the center of the country, and Antofagasta, in the north (Russ et al., 2016). It has been suggested that this difference may be due to the high urbanization rates of these regions and/or to pollution (Russ et al., 2016). Nevertheless, dementia-related mortality data should be interpreted with caution. This is because many people living with dementia are not formally diagnosed (Lang et al., 2017) and few physicians have received dementia training, thus they may underreport dementia as a cause of death (O’Neill et al., 2013; Olavarría et al., 2016; Russ et al., 2016). Since a formal diagnosis is required for it to be recorded on a death certificate, it is possible that dementia underdiagnosis is affecting the accuracy of data on dementia-related mortality (Russ et al., 2016).

## The Impact of Dementias

### Disease Burden

The DALY (disability-adjusted life years) index is used to measure and compare disease burdens in the population. It represents the sum of the years lost due to premature death and the years lost as a result of disability (Alzheimer’s Association, 2014). The World Health Organization has estimated that dementias contribute with 11.2% of the years of life with disability in people over 60 years old, which is higher than the time added by, cardiovascular diseases and cancer (Ballard et al., 2011). A global study of disease burden conducted in 2010 showed that the DALYs due to AD and other dementias rose from 5,695,000 (4,516,000–6,982,000) in 1990 to 11,349,000 (9,147,000–1,3741,000) in 2010, representing a 99.3% increase. The DALYs per 100,000 people increased from 107 (85–132) in 1990 to 165 (133–199) in 2010, representing a 53.3% increase. Globally, dementias are the 49th disease in terms of DALYs. In Central America, they occupy the 50th spot, the 62nd in Andean LA and the 26th in South America (Murray et al., 2012). In Chile, they are the fastest-growing diseases in terms of DALY causation: 200% between 1990 and 2010 (Murray et al., 2012). Likewise, dementias are the fastest-growing diseases in terms of premature death causes, rising from the 49th place in 1990 to the 17th in 2010 (Lozano et al., 2012).

### Burden of Caring

A high proportion of people with dementia need care ranging from the provision of instrumental daily living activities to full personal care and round-the-clock supervision (Sorensen et al., 2006). Therefore, dementia affects not only the patient, but also the person who supports them, which effectively doubles the number of people concerned (Ferrario et al., 2003; Georges et al., 2008). Caregivers are either formal, i.e., people who are paid to care for a patient with dementia, such as healthcare professionals, or informal, i.e., individuals who provide care and/or support to a family member, friend or neighbor who is chronically ill, frail, or who has a physical or mental disability (Slachevsky et al., 2013). In Latin American societies, patients with dementia are mainly cared for by families, and to a lesser extent by public institutions. That is, care and attention are provided by people who are physically and emotionally close to them and who have the largest responsibility in the ill person’s care, despite having no prior training and receiving no payment. Several studies have examined informal caregivers, two of which included samples with more than 200 subjects. In Chile, the CUIDEME study involved 292 family caregivers who lived mainly in Santiago, the capital city. There were more female (80%) than male caregivers. Most of them were daughters and spouses of the patients. Severe burden was reported in 63% of the caregivers, and 47% exhibited psychiatric morbidity. Burden was associated with caregiver psychiatric distress, family dysfunction, severity of neuropsychiatric symptoms, and functional disability, but neither the patient’s age, gender, nor socioeconomic status (SES) impacted burden (Slachevsky et al., 2013). In Cuba, in a descriptive study with 237 caregivers, a survey was administered to informal caregivers of elderly people with ictus and dementia, who resided in the Abel Santamaría neighborhood of Santiago de Cuba. The analysis demonstrated that 71.7% of caregivers were female and 28.3% male. Their average age was 49.83 years, and the average time devoted to the task was 42.86 months. 49.4% of the caregivers were children or partners of the people with dementia studied. 53.2% were full-time caregivers, while 46.7% worked part-time (Turtós Carbonell et al., 2016). In Bogotá, Colombia, in a descriptive study conducted with 52 informal caregivers of patients with Alzheimer’s, 82.7% of whom were female, 57.7% of the population was not aware of any support networks. 36.5% of the caregivers were 51–60 years old and 25.0% were between 41 years and 50 years old. 23.1% were under 40 years old, 9.6% were 61–70 years old, and only 5.8% were over 70. 55.8% of the caregivers were children of the patients and 30.8% were their spouses. 55.8% of the caregivers reported caring for a person with AD for 36 months and 34.6% did so between 13 months and 36 months. 36.5% of the caregivers were housewives, 26.9% were pensioners, 25% were in work, and 5.8% were unemployed. Caregiver depression, assessed with the State-Trait Depression Inventory, occurs due to taking care of a person who is ill (Cerquera Córdova et al., 2012). In Peru, in three research centers located in Lima, 92 informal caregivers were interviewed to analyze their caregiver burden with the Zarit Burden Interview (ZBI) and the Beck Depression Inventory (BDI-II). In this sample, 75% of the participants were over 55.5 years old. Most were female (81.5%) and spouses of the patient (60.87%). In addition, over 75% of them had been a caregiver for at least 1 year, 90.2% considered that their leisure time had been reduced, and 83.7% felt that their health had deteriorated. This study demonstrated that caregivers display high burden levels. Also, a multivariate analysis revealed that only the BDI-II was a solid predictor of ZBI (Custodio et al., 2014). In Brazil, at the Center for AD of the Federal University of Rio de Janeiro, 145 caregivers were interviewed to analyze three dimensions of caregiver burden: emotional exhaustion (EE), depersonalization (DP) and reduced personal accomplishment (RPA). In addition, the researchers studied the demographic characteristics of the patients’ caregivers and clinics. High levels of EE were present in 42.1% of the sample, while DP was found in 22.8%. RPA was present in 38.6% of the participating caregivers. Caregiver depression and patient delusions were the most significant predictors of EE (Truzzi et al., 2012). Similarly, in a sample of people living in Sao Paulo, 165 caregivers of patients with dementia (61 with AD, 25 with some cognitive impairment but not dementia, and 79 healthy controls) were interviewed to assess caregiver burden according to the ZBI and establish correlations with the results of the patients’ Neuropsychiatric Inventory (NPI). This study demonstrated that neuropsychiatric symptoms are significantly associated with caregiver stress (Cunha Folquitto et al., 2013).

### Economic Cost of Dementia

In 2010, the total estimated worldwide cost of dementia was US$ 817.9 billion, roughly one percent of the global gross domestic product (GDP). This cost has three components: (i) direct costs that include medical expenses (visits, tests, medication); (ii) social costs associated with paid formal caregiving by health professionals or institutionalization; and (iii) indirect costs associated with informal caregivers—family members, friends, or neighbors—who are unpaid but forgo paid jobs and thereby suffer a productivity loss. The level and composition of the cost of dementia varies widely across countries. In high-income countries, the cost is 1.2 percent of GDP and is mostly formal. In contrast, in low-income countries it is just 0.24 percent of GDP and most costs are informal (World Health Organization and Alzheimer’s Disease International, 2012; WHO, 2015). In contrast with high-income countries, informal care costs predominate in low and mid-low income countries. The costs of community care by paid social caregivers and home caregivers are practically nonexistent (Wimo et al., 2017). Only a handful of studies have focused on the cost and heterogeneity of the instruments used to assess the costs involved. Allegri et al. (2007), using a sample of 80 community-dwelling patients with dementia and 25 institutionalized patients, reported that direct costs increased according to the degree of cognitive deterioration (US$ 3420.40 in mild cases and US$ 9657.60 in severe cases) and institutionalization (US$ 3189.20 for outpatients vs. US$ 14,447.68 for institutionalized patients). Costs also differ depending on the type of dementia (US$ 5112 for VD, US$ 4625 for AD and US$ 4,924 for FTD). In Peru, Custodio et al. (2015), using a sample of 136 outpatients receiving care at a private clinic, reported an average cost of US$ 1500 per trimester for AD, US$ 1860 for FTD and US$ 1291 for VD. These costs are significantly greater than those of patients without dementia (US$ 230). Families are reported to spend a large part of their income on the care of people with dementia. In Brazil, Veras et al. (2008) reported that direct costs represent approximately 66% of family income, ranging from 75% in mild stages to 62% in severe stages, a figure that reaches 81% in the case of comorbidities such as high blood pressure and diabetes. Liu (2013) (unpublished data cited in Prince et al., 2015) reported a higher cost of public care (US$ 6750) compared to private care (US$ 1887), with both values being calculated using international dollars. In Chile, Hojman et al. (2017), using a sample of 330 informal primary caregivers, reported an average monthly cost per patient of US$ 943. Direct medical costs account for 21%, direct social costs represent 5%, and indirect costs constitute 74% of the total figure. The mean monthly cost is inversely related to SES. The monthly cost for high SES is US$ 690 and US$ 1023 for low SES. In this study, between one third and one half of the variation explained by SES is not due to the severity gradient, suggesting that SES is a key determinant in the cost of dementia, regardless of severity.

In contrast with high income countries, LA is characterized by the predominance of informal care costs (Wimo et al., 2010). Medical costs represent a relatively small percentage of the total costs of dementias. Costs associated with community care provided by paid social caregivers and home caregivers are practically nonexistent, which may be due to the lack of developed health services that can meet the needs of patients with chronic, non-transmissible diseases such as dementia (Hojman et al., 2017).

It is important to stress that the studies mentioned above have major limitations, including the fact that they employed convenience sampling and only enrolled patients diagnosed with dementias and who were being medically monitored. For this reason, these results must be extrapolated cautiously, considering that most people with dementias do not receive medical care.

## National Dementia Policies

In LA, outdated health systems have failed to provide the complex and multidisciplinary actions required by people with chronic diseases such as dementia (Bossert and Leisewitz, 2016). Following recommendations by the WHO, the Pan American Health Organization and NGOs such as AD International (World Health Organization and Alzheimer’s Disease International, 2012), several LA countries have started to develop national strategies to address the dementia crisis. Yet, much work remains to be done.

One of the countries spearheading these efforts in the region is Costa Rica, who launched in 2014 a National Plan for AD and Related Diseases (Conapam, 2014). The plan’s original objectives were transformed into seven cross-cutting principles which are guiding its implementation: (1) human rights of people with dementia; (2) empowerment and participation of people with dementia and their caregivers; (3) evidence-based practices for risk reduction and dementia care combined with research in the public and private sectors; (4) multisectoral collaboration on the response of public health to dementia; (5) health, social and community coverage for dementia; (6) equality in the response of public health in relation to dementia; and (7) care, prevention, promotion and rehabilitation treatment and development of dementia care. In 3 years, Costa Rica has implemented a number of initiatives, including community and clinical memory centers, programs, facilities and training for caregivers, acquisition of neuroimaging equipment, access to medication approved for Alzheimer’s through the social security system, among others.

In 2016, Argentina launched the National Strategic Plan for a Healthy Brain, AD and other Dementias (PAMI, 2016). The plan encompasses five key areas: improving awareness, training professionals, caregivers and family members, improving access to diagnosis and treatment, reducing risk and encouraging research. Its implementation is underway.

**Figure 1:**
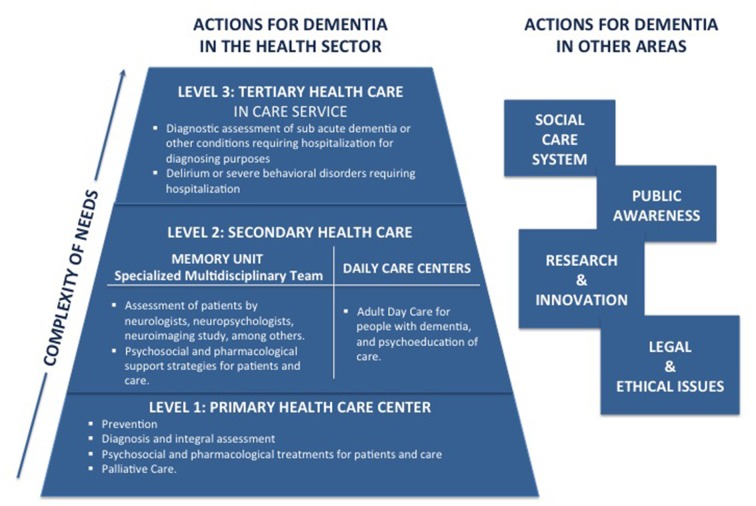
Biopsychosocial model of dementia care in the Chilean’s National Dementia Plan (adapted from Ministerio de Salud, 2015) (prepared by AS).

In 2015, the Chilean government implemented an intersectoral work group—comprised of experts in neurology, geriatrics, mental health, public policy and the civil society—to propose a National Plan for Dementia (Ministerio de Salud, 2015). The plan was submitted to public consultation and has yet to be officially launched (Gajardo and Abuseleme, 2016). Nevertheless, the plan is currently being implemented and funding has been granted for Memory Clinics in secondary health care facilities, for a pilot program for dementia in primary care facilities and for dementia care training for health professionals (Figure 1).

In addition, since 2013, the government of Chile has funded daily care facilities for dementia patients, which are an important component of the dementia plan.

Other countries, however, are lagging behind. Bolivia, for example, has approved specific laws for people with dementia, but no financial commitments have been made. In 2013, Mexico’s National Institutes of Geriatrics, Neurology and Psychiatry developed a proposal for a National Dementia Plan. Its primary aim was to promote the well-being of people affected by AD and theirs caregivers and families through the strengthening of the Mexican healthcare system and the support of other responsible institutions (Gutiérrez-Robledo and Arrieta-Cruz, 2015). However, this plan has yet to be approved by the government.

Given the challenges posed by the increasing number of people living with dementia, LA countries’ current efforts to tackle the looming dementia crisis are simply insufficient. Governments must urgently work toward closing the gap between the need for prevention, treatment and care of dementia and the actual provision of these services (World Health Organization and Alzheimer’s Disease International, 2012). National dementia policies also need to consider how key contextual factors such as poverty, inequality and limited resources, impact the health of the population and access to health services (Marmot, 2005; Baez and Ibáñez, 2016; Bossert and Leisewitz, 2016; Hojman et al., 2017).

With regard to prevention, policies must address the challenges posed by a higher prevalence of dementia in groups with less education, by the increased incidence of cardiovascular diseases, which in turn, are associated with a higher risk of dementia, and by economic and geographical barriers to access to health services (Glassman et al., 2010; Garcia-Subirats et al., 2014). Dementia prevention efforts should also be shared by other government departments. For instance, recent evidence suggests that improvement in living conditions and higher levels of formal education account for a reduced risk of dementia in later life (Wu et al., 2017). Thus, increasing access to formal education could contribute to reduce the prevalence of dementia in LA (Meng and D’arcy, 2012).

A key policy challenge is that access to appropriate support services for people with dementia is hindered by the low level of detection of dementia in LA (Lang et al., 2017). This is largely due to the social stigma associated with dementia and lack of dementia awareness both among the general public and healthcare professionals. Therefore, efforts should also focus on addressing these barriers (Romero and Ge, 2007; Maestre, 2012; Olavarría et al., 2016).

A related obstacle to dementia diagnosis is the lack of validated and standardized instruments to assess functionality and cognition in illiterate and low-educated populations and populations with diverse cultural background, such as indigenous population vs. city population (Maestre, 2012; Parra, 2014).

An important question is when in the course of the dementia syndrome it is advisable to make a diagnosis. There is currently an intense debate about the benefits and drawbacks of early diagnosis vs. timely diagnosis—the latter being defined as the access to accurate diagnosis at a time in the disease process when it can be of most benefit for the patient (Le Couteur et al., 2013). From a public health perspective, the available evidence suggests that there is no benefit in screening for cognitive impairment at the population level, and that both screening and early diagnosis could actually cause harm (Boustani et al., 2003; Le Couteur et al., 2013). In the absence of a cure for AD and other neurodegenerative dementias, timely diagnosis is, therefore, recommended (Brooker et al., 2014). Nevertheless, the concept of timely diagnosis is ill-defined in the field of dementia. For some scholars, it corresponds to a diagnosis in the presence of neuropathology, early cognitive changes and possible disability and subjective impairment, i.e., at a stage that overlaps with early diagnosis (Dubois et al., 2016). For others, timely diagnosis corresponds to either a diagnosis established at the onset of cognitive decline and disability, responding to patient and caregivers’ concerns, or to a diagnosis established in the presence of significant evidence of cognitive decline and disability (Prince et al., 2011; Brooker et al., 2014). In LA, the benefits and disadvantages associated with the diagnosis of dementia at different stages should be evaluated and a clear definition of timely diagnosis within the LA context should be sought. We argue that given the high rate of undetected dementia, the prevailing misconceptions on aging, the low awareness of dementia in the general public and health professionals, inadequate training in dementia among health professionals, and little access to specialist service, the benefit of case detection for “at risk” groups needs to be discussed (Baez and Ibáñez, 2016; Manes, 2016; Lang et al., 2017).

Concerning treatment and care, most LA countries have outdated health systems which are not currently able to offer the resource-intensive and multidisciplinary programs that people with dementia and their caregivers require (Bossert and Leisewitz, 2016). The capacity to develop integrated health and social care services for patients with dementia continues to challenge both developed and developing economies and will certainly be paramount to the success of public policies attempting to address the rising tide of dementia. Meanwhile, raising public awareness about dementia and ensuring that those living with these illnesses are treated with respect and dignity should be a priority (Gajardo and Abuseleme, 2016; Slachevsky and Gajardo, in press).

## Conclusions

The increase in the prevalence of dementia and the resulting economic and societal impact, are growing concerns in LA. Yet, only a handful of epidemiological studies examining the prevalence of risk and dementia types, and protective factors, have been conducted in the region. Moreover, there is scarce information regarding the socio-sanitary conditions of people with dementia, their environment and associated costs. Among other issues, there is no data evaluating the impact of diagnosis and access to socio-sanitary support services on the evolution of the disease. From a public health perspective, the vast and rapid increase in the number of people with dementia due to demographic and health changes, warrants the prioritization of both dementia prevention policies long-term strategies to provide care for people living with dementia (Sousa et al., 2010).

Finally, it should be noted that although LA can be considered as a whole, its levels of development are heterogeneous. Risk factors associated with dementia, as well as its social impact varies between countries and within each country. Therefore, research and public policies in this field require consideration of the similarities that characterize the LA region, as well as its differences and particularities.

## Author Contributions

All the authors developed the study concept, drafted the manuscript and approved its final version.

## Conflict of Interest Statement

The authors declare that the research was conducted in the absence of any commercial or financial relationships that could be construed as a potential conflict of interest.
